# Pricing quanto options with market liquidity risk

**DOI:** 10.1371/journal.pone.0292324

**Published:** 2023-09-28

**Authors:** Rui Gao, Yanfei Bai

**Affiliations:** 1 School of Statistics and Mathematics, Shandong University of Finance and Economics, Jinan, China; 2 School of Insurance, Shandong University of Finance and Economics, Jinan, China; Public Library of Science, UNITED STATES

## Abstract

This paper investigates the pricing problem of quanto options with market liquidity risk using the Bayesian method. The increasing volatility of global financial markets has made liquidity risk a significant factor that should be taken into consideration while evaluating option prices. To address this issue, we first derive the pricing formula for quanto options with liquidity risk. Next, we construct a likelihood function to conduct posterior inference on model parameters. We then propose a numerical algorithm to conduct statistical inferences on the option prices based on the posterior distribution. This proposed method considers the impact of parameter uncertainty on option prices. Finally, we conduct a comparison between the Bayesian method and traditional estimation methods to examine their validity. Empirical results show that our proposed method is feasible for pricing and predicting quanto options with liquidity risk, particularly for parameter estimations with a small sample size.

## 1 Introduction

Quanto options are increasingly becoming an essential tool for financial investment and risk management as financial globalization progresses. Quanto option is a multi-asset option whose value depends on the underlying asset in one currency, but the payoff settled in another currency, enabling the holder to manage the multinational risks from diverse financial markets.

With the development of option pricing theory, many extended quanto option pricing models have been proposed based on the Black-Scholes [1] model. To better capture market characteristics, such as volatility smile, heavy tails, skewness, and jump, existing literature incorporates these features into quanto option pricing models, including GARCH models, stochastic volatility models, jump-diffusion models, etc. More related research refers to [2–5]. Moreover, Teng et al. [6] assumed that the correlation between the underlying asset and currency exchange rate is dynamic, and they found that this dynamic correlation had a significant impact on the quanto option pricing. Battauz et al. [7] studied the optimal exercise policies of American quanto options by using a parsimonious diffusive model, which further enriched the pricing theory of quanto options. Recently, Lee et al. [8] studied partial quanto lookback options and proposed an approach to evaluating the option. Their pricing formula makes quanto lookback options cheaper.

The continuous international trade war and COVID-19 have increased the volatility of financial markets. As a result of increased market volatility, investors are increasingly concerned with market liquidity when managing their financial assets. Moreover, researchers have found that the effect of market liquidity should be considered in option pricing. Brunetti and Caldarera [9] established the liquidity discount factor and liquidity-adjusted asset pricing model for capturing the characteristics of stocks in an imperfectly liquid market. On the foundation of this model, Li et al. [10] further proposed the quanto option pricing model with liquidity adjustment. They found that considering stock liquidity in the option pricing model can better fit market data. Gao et al. [11] studied the exchange option pricing problem with market liquidity in an incomplete market. Pasricha et al. [12] have reviewed the relevant literature and classified this type of research into two categories, i.e., stock-specific liquidity and market-wide liquidity. They developed a theoretical framework to consider liquidity risk in the pricing of European options based on market-wide liquidity. For the former, some scholars found that trading in stock could have an influence on its price and studied the effect of stock-specific liquidity on option pricing, see, for example, [13, 14]. For the latter, some empirical results demonstrated that the commonality in liquidity affects stock returns and the option pricing, see, for example, [15–19]. Recently, Pasricha and He [20] employed an Ornstein–Uhlenbeck process to model market liquidity risk and studied the effect of stochastic liquidity on exchange options. These studies demonstrate that the impact of market liquidity risk on option pricing should be considered, which can improve the pricing performance.

There have been many extended studies on the theoretical pricing model for quanto options. However, there are few studies on parameter estimations of the quanto option pricing model in existing literature, especially in illiquid markets. The accuracy of parameter estimation directly affects the performance of option pricing models. Due to the lack of market data on quanto options, traditional estimation methods that rely on a large number of sample data, such as Maximum Likelihood Estimation and optimization method, may no longer be effective for quanto option pricing models. However, for complicated models, the Bayesian statistical method, which can fully consider prior information and parameter uncertainty, performs better in parameter inference and prediction. Therefore, some researchers suggest applying the Bayesian method to estimate model parameters; see, e.g., [21–25]. Karolyi [21] considered the impact of the randomness of volatility on stock returns and proposed an approach to evaluating European call options under the Bayesian framework. Rombouts and Stentoft [22] introduced an approach to conducting posterior inference on European option price and demonstrated that the Bayesian method performs better than traditional methods when sample data is small. Gao et al. [26] introduced an approach to conducting posterior inference on the European call option pricing model in an imperfectly liquid market. Recently, Hu et al. [27] proposed a new semi-parametric nonlinear volatility model to capture stock returns and they recommended a Bayesian sampling algorithm for estimating the model parameters.

However, research on the quanto option pricing model with market liquidity using the Bayesian method is rare. Considering the advantages of Bayesian statistics in parameter estimations for small samples, we propose an alternative approach to evaluating quanto options with liquidity risk under the Bayesian framework. Based on posterior distributions, we perform statistical inferences on model parameters and the option price by the Markov chain Monte Carlo (MCMC) numerical algorithm. In the numerical experiment, the liquidity is defined as the ability of an asset to trade any amount of securities quickly at the market price without additional transaction cost. We adopt the commonly used liquidity measure to capture the liquidity risk. The liquidity measure is defined as stock return divided by dollar trading volume (hereafter RDV). The RDV measure can be used to describe a sudden down or up in a stock price. This measure is negative when the price is down and positive when it is up. The RDV measure is zero when the market is perfectly liquid; refer to [15] for more details.

The main work of this paper is as follows. The pricing problem of the quanto option is studied using the Bayesian method in an incomplete market. This paper provides an alternative approach to evaluating quanto options with liquidity risk. First, an explicit expression of the quanto option price with liquidity risk is derived from a mathematical perspective. Second, under the Bayesian framework, an estimation approach is proposed to conduct statistical inferences on model parameters and the quanto option price. We account for the randomness of model parameters as well as the randomness of the correlation coefficient between the underlying stock and the exchange rate. Moreover, the quanto option price can be predicted by the posterior density. A comparison between the proposed method and the traditional estimation method is conducted to examine the validity.

This paper is different from the existing literature in the following aspects. First, we derive the closed-form pricing formula of the quanto option with liquidity risk in an alternative way. Second, we propose a Bayesian approach to estimate model parameters. We consider the effects of parameter uncertainty and the correlation coefficient randomness on the quanto option price. Moreover, we investigate the statistical properties of the quanto option prices based on posterior distributions by an MCMC numerical algorithm. Unlike traditional methods that usually provide only a point estimate, we offer more statistical characteristics about option prices from a probabilistic perspective. These statistical characteristics can provide investors with more information to make better decisions.

The remainder of this article is as follows. Section 2 describes the stock price process in an imperfectly liquid market and deduces the pricing formula for the quanto option with different payoffs. Section 3 introduces the posterior inferences on parameters and the quanto option price. Section 4 conducts an empirical analysis. Section 5 is the conclusion.

## 2 Quanto option pricing model with liquidity risk

In this article, we investigate the pricing problem of a European quanto option in an incomplete market, where the underlying asset is an imperfectly liquid foreign stock. With the quanto option as an example, we provide an approach to studying the pricing of multi-asset options with market liquidity risk.

### 2.1 Dynamics of the foreign stock price with liquidity risk

To investigate the effect of stock liquidity risk on quanto options, we adopt the liquidity-adjusted asset pricing model proposed by [9] to model the foreign stock price dynamics

$$\begin{array}{c} \frac{dS(t)}{S(t)}=\left(\mu +\xi \omega \left(t\right)+\frac{1}{2}\xi^{2}\omega^{2}\left(t\right)\right)dt+\xi \omega \left(t\right)dW_{L}^{P}\left(t\right)+\lambda dW_{I}^{P}\left(t\right), \end{array}$$ (1)

where *ω*(*t*) denotes the liquidity level at time *t* ∈ [0, *T*], *ω*(*t*) > 0 (*ω*(*t*) < 0) shows that the market is in shortage (surplus), *ω*(*t*) = 0 indicates a perfectly liquid market. *ξ* > 0 denotes the sensitivity of stock price *S*(*t*) to liquidity level *ω*(*t*), and λ is a part of volatility. *L*(*t*) and *I*(*t*) respectively, represent the processes of the liquidity discount factor and the information following

$$\begin{array}{c} \frac{dL(t)}{L(t)}=\left(\frac{1}{2}\xi^{2}\omega^{2}\left(t\right)-\xi \omega \left(t\right)\right)dt-\xi \omega \left(t\right)dW_{L}^{P}\left(t\right), \end{array}$$

and

$$\begin{array}{c} \frac{dI(t)}{I(t)}=\mu_{I}dt+\sigma_{I}dW_{I}^{P}\left(t\right), \end{array}$$

where $W_{L}^{P}\left(t\right)$ and $W_{I}^{P}\left(t\right)$ are independent Brownian motions under physical measure P. Similarly to [10, 12, 20], we employ the liquidity discount factor *L*(*t*) to capture the effect of the liquidity risk. *L*(*t*) is a function of the liquidity level *ω*(*t*) and a parameter *ξ* representing the sensitivity of the stock price to the liquidity level.

### 2.2 Quanto option model with liquidity risk

In the paper, we consider a quanto option on an imperfectly liquid stock whose price dynamics are given by Eq (1). Supposing that the price processes of the foreign stock and exchange rate are followed by

$$\begin{array}{c} \left\{ \begin{array}{cc} dS\left(t\right)=\left(\mu +\xi \omega \left(t\right)+\frac{1}{2}\xi^{2}\omega^{2}\left(t\right)\right)S(t)dt+\xi \omega \left(t\right)S(t)dW_{L}^{P}\left(t\right)+\lambda S(t)dW_{I}^{P}\left(t\right), & \\ dF\left(t\right)=\mu_{F}F(t)dt+\sigma_{F}F(t)dW_{F}^{P}\left(t\right), \end{array}\right. \end{array}$$ (2)

where *ρ* denotes the correlation coefficient between *S*(*t*) and *F*(*t*), i.e., $dW_{F}^{P}\left(t\right)dW_{I}^{P}\left(t\right)=\rho$, and $dW_{F}^{P}\left(t\right)dW_{L}^{P}\left(t\right)=0$. Therefore, the Brownian motion $W_{I}^{P}\left(t\right)$ can be represented by $W_{I}^{P}\left(t\right)=\rho W_{F}^{P}\left(t\right)+\sqrt{1-\rho^{2}}W^{P}\left(t\right)$, where $W_{L}^{P}\left(t\right)$ and $W_{F}^{P}\left(t\right)$ are independent of $W^{P}\left(t\right)$.

Then, the dynamics of the foreign stock price can be rewritten by

$$\begin{array}{c} \frac{dS(t)}{S(t)}=\left(\mu +\xi \omega \left(t\right)+\frac{1}{2}\xi^{2}\omega^{2}\left(t\right)\right)dt+\xi \omega \left(t\right)dW_{L}^{P}\left(t\right)+\lambda \rho dW_{F}^{P}\left(t\right)+\lambda \sqrt{1-\rho^{2}}dW^{P}\left(t\right). \end{array}$$

Denote

$$\sqrt{\xi^{2}\omega^{2}\left(t\right)+\lambda^{2}\left(1-\rho^{2}\right)}d{\tilde{W}}^{P}\left(t\right)=\xi \omega \left(t\right)dW_{L}^{P}\left(t\right)+\lambda \sqrt{1-\rho^{2}}dW^{P}\left(t\right),$$

thus, we have

$$\begin{array}{c} \frac{dS(t)}{S(t)}=\left(\mu +\xi \omega \left(t\right)+\frac{1}{2}\xi^{2}\omega^{2}\left(t\right)\right)dt+\sqrt{\xi^{2}\omega^{2}\left(t\right)+\lambda^{2}\left(1-\rho^{2}\right)}d{\tilde{W}}^{P}\left(t\right)+\rho \lambda dW_{F}^{P}\left(t\right). \end{array}$$

For evaluating the quanto option by martingale pricing theory, we need to find the equivalent martingale measure. Similarly to [10], by multidimensional Girsanov theorem, we deduce the equivalent martingale measure Q defined by the Radon-Nikodym derivative

$$\begin{array}{c} \frac{dQ}{dP}=\text{exp}\{\sum_{i=1}^{2}[\int_{0}^{t}-\gamma_{i}\left(u\right)dW_{i}^{P}\left(u\right)-\frac{1}{2}\int_{0}^{t}\gamma_{i}^{2}\left(u\right)du]\}, \end{array}$$

where $W_{1}^{P}\left(t\right)={\tilde{W}}^{P}\left(t\right)$, $W_{2}^{P}\left(t\right)=W_{F}^{P}\left(t\right)$, and

$$\begin{array}{c} \left\{ \begin{array}{c} \;\gamma_{1}\left(t\right)=\frac{\mu_{F}+\mu +\xi \omega \left(t\right)+\frac{1}{2}\xi^{2}\omega^{2}\left(t\right)+\rho \lambda \sigma_{F}-r_{d}-\left(\sigma_{F}+\rho \lambda \right)\gamma_{2}\left(t\right)}{\sqrt{\xi^{2}\omega^{2}\left(t\right)+\lambda^{2}\left(1-\rho^{2}\right)}},\\ \;\gamma_{2}\left(t\right)=\frac{\mu_{F}+r_{f}-r_{d}}{\sigma_{F}}, \end{array}\right. \end{array}$$

$F_{t}$ denotes the filtration. Then, we deduce the corresponding Brownian motions under measure Q following

$$\begin{array}{c} d{\tilde{W}}^{Q}\left(t\right)=d{\tilde{W}}^{P}\left(t\right)+\gamma_{1}\left(t\right)dt,\\ dW_{F}^{Q}\left(t\right)=dW_{F}^{P}\left(t\right)+\gamma_{2}\left(t\right)dt, \end{array}$$

where $d{\tilde{W}}^{Q}\left(t\right)dW_{F}^{Q}\left(t\right)=0$.

Therefore, the price processes of the foreign stock and exchange rate are followed by

$$\begin{array}{c} \left\{ \begin{array}{c} \;\frac{dS(t)}{S(t)}=\left(r_{f}-\rho \lambda \sigma_{F}\right)dt+\sqrt{\xi^{2}\omega^{2}\left(t\right)+\lambda^{2}\left(1-\rho^{2}\right)}d{\tilde{W}}^{Q}\left(t\right)+\rho \lambda dW_{F}^{Q}\left(t\right),\\ \;\frac{dF(t)}{F(t)}=\left(r_{d}-r_{f}\right)dt+\sigma_{F}dW_{F}^{Q}\left(t\right), \end{array}\right. \end{array}$$ (3)

under domestic risk neutral measure Q.

By Ito formula, we have

$$d\;\text{ln}\;S\left(t\right)=\left(r_{f}-\rho \lambda \sigma_{F}-\frac{1}{2}\xi^{2}\omega^{2}\left(t\right)-\frac{1}{2}\lambda^{2}\right)dt+\sqrt{\xi^{2}\omega^{2}\left(t\right)+\lambda^{2}\left(1-\rho^{2}\right)}d{\tilde{W}}^{Q}\left(t\right)+\rho \lambda dW_{F}^{Q}\left(t\right),$$ (4)

$$d\;\text{ln}\;F(t)=\left(r_{d}-r_{f}-\frac{1}{2}\sigma_{F}^{2}\right)dt+\sigma_{F}dW_{F}^{Q}\left(t\right).$$ (5)

Next, we deduce the theoretical pricing model of the quanto option in an imperfectly liquid market.

### 2.3 Theoretical pricing model of quanto options with liquidity risk

Similarly to [10], we consider four different types of payoffs for the quanto option on an imperfectly liquid stock. Assuming that the dynamics of the underlying asset are followed by Eq (2).

According to the martingale pricing theory, we deduce the pricing formulas of quanto options with four different payoffs under the domestic martingale measure Q.

**Theorem 1**. *Suppose the underlying foreign asset is an imperfectly liquid stock* *S*(*t*) *defined by* Eq (1), *then the time-t price of the floating exchange rate foreign stock quanto call option struck in foreign currency at maturity T with payoff F*(*T*) max{*S*(*T*) − *K*_*f*_, 0} *is*

$$\begin{array}{c} V_{1}\left(S\left(t\right),F\left(t\right),\omega \left(t\right),\lambda ,\xi \right)=F\left(t\right)\left[S\left(t\right)\Phi \left(d_{1}^{\left(1\right)}\right)-K_{f}e^{-r_{f}\tau }\Phi \left(d_{2}^{\left(1\right)}\right)\right], \end{array}$$ (6)

*where* *τ* = *T* − *t*, *ω*(*t*) *is liquidity level*, λ, *ξ* *are defined as previously, and*

$$\begin{array}{cc} & d_{1}^{\left(1\right)}=\frac{\text{ln}\;\frac{S(t)}{K_{f}}+\left[\left(r_{f}+\frac{1}{2}\lambda^{2}\right)\tau +\frac{1}{2}\xi^{2}\int_{t}^{T}\omega^{2}\left(u\right)du\right]}{\sqrt{\lambda^{2}\tau +\xi^{2}\int_{t}^{T}\omega^{2}\left(u\right)du}},\\ & d_{2}^{\left(1\right)}=d_{1}^{\left(1\right)}-\sqrt{\lambda^{2}\tau +\xi^{2}\int_{t}^{T}\omega^{2}\left(u\right)du}\;. \end{array}$$

*Proof*. By the martingale pricing theory, the price of the floating exchange rate foreign stock quanto call option struck in foreign currency can be given by

$$\begin{array}{c} V_{1}\left(S\left(t\right),F\left(t\right),\omega \left(t\right),\lambda ,\xi \right)=e^{-r_{d}\tau }E^{Q}\left[F\left(T\right)\;\text{max}\left\{S\left(T\right)-K_{f},0\right\}|F_{t}\right], \end{array}$$ (7)

where $E^{Q}\left[\cdot \right]$ is the expectation operator under domestic risk-neutral martingale measure Q.

From Eq (5), we have

$$\begin{array}{c} F\left(t\right)=F\left(0\right)\;\text{exp}\{\left(r_{d}-r_{f}-\frac{1}{2}\sigma_{F}^{2}\right)t+\sigma_{F}W_{F}^{Q}\left(t\right)\}, \end{array}$$

and denote the equivalent martingale measure by $Q_{1}$ defined by

$$\begin{array}{c} \frac{dQ_{1}}{dQ}=\text{exp}\left\{-\frac{1}{2}\sigma_{F}^{2}t+\sigma_{F}W_{F}^{Q}\left(t\right)\right\}. \end{array}$$

Then, formula (7) can be rewritten as

$$\begin{array}{c} V_{1}\left(S\left(t\right),F\left(t\right),\omega \left(t\right),\lambda ,\xi \right)\\ =e^{-r_{d}\tau }E^{Q}\left[F\left(t\right)e^{\left(r_{d}-r_{f}\right)\left(T-t\right)}\frac{dQ_{1}}{dQ}\text{max}\;\left\{S\left(T\right)-K_{f},0\right\}|F_{t}\right],\\ =F\left(t\right)e^{-r_{f}\tau }E^{Q_{1}}\left[S\left(T\right)I_{\left\{K_{f}<S\left(T\right)\right\}}|F_{t}\right]-F\left(t\right)e^{-r_{f}\left(T-t\right)}K_{f}E^{Q_{1}}\left[I_{\left\{K_{f}<S\left(T\right)\right\}}|F_{t}\right], \end{array}$$ (8)

where *I*_{}_ denotes the indicator function.

Under equivalent martingale measure $Q_{1}$, we obtain

$$d\;\text{ln}\;S\left(t\right)=\left(r_{f}-\frac{1}{2}\xi^{2}\omega^{2}\left(t\right)-\frac{1}{2}\lambda^{2}\right)dt+\sqrt{\xi^{2}\omega^{2}\left(t\right)+\lambda^{2}\left(1-\rho^{2}\right)}d{\tilde{W}}^{Q_{1}}\left(t\right)+\rho \lambda dW_{F}^{Q_{1}}\left(t\right),$$

where ${\tilde{W}}^{Q_{1}}\left(t\right)$ and $W_{F}^{Q_{1}}\left(t\right)$ are independent standard Brownian motions under measure $Q_{1}$ satisfying

$$\begin{array}{c} d{\tilde{W}}^{Q_{1}}\left(t\right)=d{\tilde{W}}^{Q}\left(t\right),\;dW_{F}^{Q_{1}}\left(t\right)=dW_{F}^{Q}\left(t\right)-\sigma_{F}dt. \end{array}$$

Thus, we get

$$\begin{array}{cc} S(T) & =S\left(t\right)\;\text{exp}\;\{\int_{t}^{T}\left(r_{f}-\frac{1}{2}\xi^{2}\omega^{2}\left(u\right)-\frac{1}{2}\lambda^{2}\right)du+\int_{t}^{T}\sqrt{\xi^{2}\omega^{2}\left(u\right)+\lambda^{2}\left(1-\rho^{2}\right)}d{\tilde{W}}^{Q_{1}}\left(u\right)\\ & +\int_{t}^{T}\rho \lambda dW_{F}^{Q_{1}}\left(u\right)\}. \end{array}$$

Therefore, the second expectation expression on the right side of Eq (8) can be given by

$$\begin{array}{cc} E^{Q_{1}}\left[I_{\left\{K_{f}<S\left(T\right)\right\}}|F_{t}\right] & ={\text{Pr}}^{Q_{1}}\left(K_{f}<S\left(T\right)|F_{t}\right)\\ & =\Phi (\frac{\text{ln}\;\frac{S(t)}{K_{f}}+\left[\left(r_{f}-\frac{1}{2}\lambda^{2}\right)\tau -\frac{1}{2}\xi^{2}\int_{t}^{T}\omega^{2}\left(u\right)du\right]}{\sqrt{\lambda^{2}\tau +\xi^{2}\int_{t}^{T}\omega^{2}\left(u\right)du}}) \end{array}$$

Next, we derive the first expectation in Eq (8). By Girsanov theorem, we obtain an equivalent martingale measure $Q_{2}$ defined by

$$\begin{array}{c} \frac{dQ_{2}}{dQ_{1}}=\text{exp}\{-\frac{1}{2}\int_{0}^{t}\left(\xi^{2}\omega^{2}\left(u\right)+\lambda^{2}\right)du+\int_{0}^{t}\sqrt{\xi^{2}\omega^{2}\left(u\right)+\lambda^{2}}dW_{3}^{Q_{1}}\left(u\right)\}. \end{array}$$

where $\sqrt{\xi^{2}\omega^{2}\left(t\right)+\lambda^{2}}dW_{3}^{Q_{1}}\left(t\right)≜\sqrt{\xi^{2}\omega^{2}\left(t\right)+\lambda^{2}\left(1-\rho^{2}\right)}d{\tilde{W}}^{Q_{1}}\left(t\right)+\rho \lambda dW_{F}^{Q_{1}}\left(t\right)$.

Based on Girsanov theorem, we obtain the standard Brownian motions $W_{3}^{Q_{2}}\left(t\right)$ under measure $Q_{2}$ satisfying

$$\begin{array}{c} dW_{3}^{Q_{2}}\left(t\right)=d{\tilde{W}}^{Q_{1}}\left(t\right)+\sqrt{\xi^{2}\omega^{2}\left(t\right)+\lambda^{2}}dt, \end{array}$$

Based on Ito formula, under measure $Q_{2}$, we derive

$$\begin{array}{c} d\;\text{ln}\;S\left(t\right)=\left(r_{f}+\frac{1}{2}\xi^{2}\omega^{2}\left(t\right)+\frac{1}{2}\lambda^{2}\right)dt+\sqrt{\xi^{2}\omega^{2}\left(t\right)+\lambda^{2}}dW_{3}^{Q_{2}}\left(t\right) \end{array}$$ (9)

Thus, the first expectation is given by

$$\begin{array}{cc} E^{Q_{1}}\left[S\left(T\right)I_{\left\{K_{f}<S\left(T\right)\right\}}|F_{t}\right] & =E^{Q_{1}}\left[e^{r_{f}\tau }S\left(t\right)\frac{dQ_{2}}{dQ_{1}}I_{\left\{K_{f}<S\left(T\right)\right\}}|F_{t}\right]\\ & =e^{r_{f}\tau }S\left(t\right)E^{Q_{2}}\left[I_{\left\{K_{f}<S\left(T\right)\right\}}|F_{t}\right]\\ & =e^{r_{f}\tau }S\left(t\right){\text{Pr}}^{Q_{2}}\left(K_{f}<S\left(T\right)|F_{t}\right)\\ & =e^{r_{f}\tau }S\left(t\right)\Phi \left(d_{1}^{\left(1\right)}\right), \end{array}$$

where $d_{1}^{\left(1\right)}=\frac{\text{ln}\;\frac{S(t)}{K_{f}}+\left[\left(r_{f}+\frac{1}{2}\lambda^{2}\right)\tau +\frac{1}{2}\xi^{2}\int_{t}^{T}\omega^{2}\left(u\right)du\right]}{\sqrt{\lambda^{2}\tau +\xi^{2}\int_{t}^{T}\omega^{2}\left(u\right)du}}$.

Therefore, the price of the quanto call option with liquidity risk is

$$\begin{array}{c} V_{1}\left(S\left(t\right),F\left(t\right),\omega \left(t\right),\lambda ,\xi \right)=F\left(t\right)\left[S\left(t\right)\Phi \left(d_{1}^{\left(1\right)}\right)-K_{f}e^{-r_{f}\tau }\Phi \left(d_{2}^{\left(1\right)}\right)\right]. \end{array}$$

**Theorem 2**. *Suppose the underlying foreign asset is an imperfectly liquid stock* *S*(*t*) *defined by* Eq (1), *then the time*-*t* *price of the quanto call option struck in domestic currency at maturity* *T* *with payoff* max{*F*(*T*)*S*(*T*) − *K*_*d*_, 0} *is*

$$\begin{array}{c} V_{2}\left(S\left(t\right),F\left(t\right),\omega \left(t\right),\lambda ,\xi ,\rho ,\sigma_{F}\right)=F\left(t\right)S\left(t\right)\Phi \left(d_{1}^{\left(2\right)}\right)-K_{d}e^{-r_{d}\tau }\Phi \left(d_{2}^{\left(2\right)}\right), \end{array}$$ (10)

*where* *τ* = *T* − *t*, *ω*(*t*) *is liquidity level*, λ, *ξ* *are defined as previously, and*

$$\begin{array}{c} d_{1}^{\left(2\right)}=\frac{\text{ln}\;\frac{F(t)S(t)}{K_{d}}+\left[\left(r_{d}+\frac{1}{2}\lambda^{2}+\rho \lambda \sigma_{F}+\frac{1}{2}\sigma_{F}^{2}\right)\tau +\frac{1}{2}\xi^{2}\int_{t}^{T}\omega^{2}\left(u\right)du\right]}{\sqrt{\left(\lambda^{2}+2\rho \lambda \sigma_{F}+\sigma_{F}^{2}\right)\tau +\xi^{2}\int_{t}^{T}\omega^{2}\left(u\right)du}},\\ d_{2}^{\left(2\right)}=d_{1}^{\left(2\right)}-\sqrt{\left(\lambda^{2}+2\rho \lambda \sigma_{F}+\sigma_{F}^{2}\right)\tau +\xi^{2}\int_{t}^{T}\omega^{2}\left(u\right)du}\;. \end{array}$$

*Proof*. By martingale pricing theory, we have

$$\begin{array}{cc} V_{2}\left(S\left(t\right),F\left(t\right),\omega \left(t\right),\lambda ,\xi ,\rho \right) & =e^{-r_{d}\tau }E^{Q}\left[\text{max}\;\left\{F\left(T\right)S\left(T\right)-K_{d},0\right\}|F_{t}\right]\\ & =e^{-r_{d}\tau }E^{Q}\left[\hat{S}\left(T\right)I_{\left\{K_{d}<\hat{S}\left(T\right)\right\}}|F_{t}\right]-e^{-r_{d}\tau }E^{Q}\left[K_{d}I_{\left\{K_{d}<\hat{S}\left(T\right)\right\}}|F_{t}\right], \end{array}$$ (11)

where $\hat{S}\left(T\right)=F\left(T\right)S\left(T\right)$.

According to Girsanov theorem, from Eq (13), we derive

$$\begin{array}{cc} d\;\text{ln}\hat{S}\left(t\right) & =(r_{d}-\frac{1}{2}\xi^{2}\omega^{2}\left(t\right)-\frac{1}{2}\lambda^{2}-\frac{1}{2}\sigma_{F}^{2}-\rho \lambda \sigma_{F})dt\\ & \;\;\;\;+\sqrt{\xi^{2}\omega^{2}\left(t\right)+\lambda^{2}\left(1-\rho^{2}\right)}d{\tilde{W}}^{Q}\left(t\right)+\left(\sigma_{F}+\rho \lambda \right)dW_{F}^{Q}\left(t\right). \end{array}$$ (12)

Then, we have

$$\begin{array}{cc} \hat{S}\left(T\right) & =\hat{S}\left(t\right)\;\text{exp}\;\{\int_{t}^{T}\left(r_{d}-\frac{1}{2}\xi^{2}\omega^{2}\left(u\right)-\frac{1}{2}\lambda^{2}-\frac{1}{2}\sigma_{F}^{2}-\rho \lambda \sigma_{F}\right)du\\ & \;\;\;\;\;\;+\int_{t}^{T}\sqrt{\xi^{2}\omega^{2}\left(u\right)+\lambda^{2}\left(1-\rho^{2}\right)}d{\tilde{W}}^{Q}\left(u\right)+\int_{t}^{T}\left(\sigma_{F}+\rho \lambda \right)dW_{F}^{Q}\left(t\right)\}. \end{array}$$ (13)

Denote the equivalent martingale measure by $Q_{1}$ defined by the following Radon-Nikodym derivative

$$\begin{array}{cc} \frac{dQ_{1}}{dQ}= & \text{exp}\{-\frac{1}{2}\int_{t}^{T}\left(\xi^{2}\omega^{2}\left(u\right)+\lambda^{2}+\sigma_{F}^{2}+2\rho \lambda \sigma_{F}\right)du\\ & \;\;\;\;\;\;\;\;+\int_{t}^{T}\sqrt{\xi^{2}\omega^{2}\left(u\right)+\lambda^{2}+\sigma_{F}^{2}+2\rho \lambda \sigma_{F}}dW_{4}^{Q}\left(u\right)\}, \end{array}$$ (14)

where

$$\begin{array}{cc} \sqrt{\xi^{2}\omega^{2}\left(u\right)+\lambda^{2}+\sigma_{F}^{2}+2\rho \lambda \sigma_{F}}dW_{4}^{Q}\left(u\right)≜ & \sqrt{\xi^{2}\omega^{2}\left(u\right)+\lambda^{2}\left(1-\rho^{2}\right)}d{\tilde{W}}^{Q}\left(u\right)\\ & +\int_{t}^{T}\left(\sigma_{F}+\rho \lambda \right)dW_{F}^{Q}\left(t\right). \end{array}$$

Substituting Eqs (13) and (14) into Eq (11), then we obtain

$$\begin{array}{c} V_{2}\left(S\left(t\right),F\left(t\right),\omega \left(t\right),\lambda ,\xi ,\rho \right)\\ =e^{-r_{d}\tau }E^{Q}\left[\hat{S}\left(T\right)I_{\left\{K_{d}<\hat{S}\left(T\right)\right\}}|F_{t}\right]-e^{-r_{d}\tau }E^{Q}\left[K_{d}I_{\left\{K_{d}<\hat{S}\left(T\right)\right\}}|F_{t}\right],\\ =e^{-r_{d}\tau }E^{Q}\left[e^{r_{d}\tau }\hat{S}\left(t\right)\frac{dQ_{1}}{dQ}I_{\left\{K_{d}<\hat{S}\left(T\right)\right\}}|F_{t}\right]-e^{-r_{d}\tau }E^{Q}\left[K_{d}I_{\left\{K_{d}<\hat{S}\left(T\right)\right\}}|F_{t}\right]\\ =\hat{S}\left(t\right)E^{Q_{1}}\left[I_{\left\{K_{d}<\hat{S}\left(T\right)\right\}}|F_{t}\right]-e^{-r_{d}\tau }K_{d}E^{Q}\left[I_{\left\{K_{d}<\hat{S}\left(T\right)\right\}}|F_{t}\right],\\ =\hat{S}\left(t\right){\text{Pr}}^{Q_{1}}\left(K_{d}<\hat{S}\left(T\right)|F_{t}\right)-e^{-r_{d}\tau }K_{d}{\text{Pr}}^{Q}\left(K_{d}<\hat{S}\left(T\right)|F_{t}\right) \end{array}$$

Based on Girsanov theorem and probability theory, we derive

$${\text{Pr}}^{Q_{1}}\left(K_{d}<\hat{S}\left(T\right)|F_{t}\right)=\Phi \left(d_{1}^{\left(2\right)}\right),\;\;{\text{Pr}}^{Q}\left(K_{d}<\hat{S}\left(T\right)|F_{t}\right)=\Phi \left(d_{2}^{\left(2\right)}\right),$$

where

$$\begin{array}{c} d_{1}^{\left(2\right)}=\frac{\text{ln}\;\frac{F(t)S(t)}{K_{d}}+\left[\left(r_{d}+\frac{1}{2}\lambda^{2}+\rho \lambda \sigma_{F}+\frac{1}{2}\sigma_{F}^{2}\right)\tau +\frac{1}{2}\xi^{2}\int_{t}^{T}\omega^{2}\left(u\right)du\right]}{\sqrt{\left(\lambda^{2}+2\rho \lambda \sigma_{F}+\sigma_{F}^{2}\right)\tau +\xi^{2}\int_{t}^{T}\omega^{2}\left(u\right)du}},\\ d_{2}^{\left(2\right)}=d_{1}^{\left(2\right)}-\sqrt{\left(\lambda^{2}+2\rho \lambda \sigma_{F}+\sigma_{F}^{2}\right)\tau +\xi^{2}\int_{t}^{T}\omega^{2}\left(u\right)du}\;. \end{array}$$

Therefore, the price of the quanto call option with liquidity risk is

$$V_{2}\left(S\left(t\right),F\left(t\right),\omega \left(t\right),\lambda ,\xi ,\rho ,\sigma_{F}\right)=F\left(t\right)S\left(t\right)\Phi \left(d_{1}^{\left(2\right)}\right)-K_{d}e^{-r_{d}\tau }\Phi \left(d_{2}^{\left(2\right)}\right).$$

**Theorem 3**. *Suppose the underlying foreign asset is an imperfectly liquid stock S*(*t*) *defined by* Eq (1), *then the time*-*t* *price of the fixed exchange rate foreign equity call at maturity T with payoff* *F*_0_ max{*S*(*T*) − *K*_*f*_, 0} *is*

$$V_{3}\left(S\left(t\right),F\left(t\right),\omega \left(t\right),\lambda ,\xi ,\rho ,\sigma_{F}\right)=F_{0}e^{-r_{d}\tau }\left[S\left(t\right)e^{(r_{f}-\rho \lambda \sigma_{F})\tau }\Phi \left(d_{1}^{\left(3\right)}\right)-K_{f}\Phi \left(d_{2}^{\left(3\right)}\right)\right],$$ (15)

*where F*_0_ *is the predetermined fixed exchange rate*, *τ* = *T* − *t*, *and* *ω*(*t*) *is the liquidity level*, λ, *ξ* *are defined as previously, and*

$$\begin{array}{c} d_{1}^{\left(3\right)}=\frac{\text{ln}\;\frac{S(t)}{K_{f}}+\left[\left(r_{f}+\frac{1}{2}\lambda^{2}-\rho \lambda \sigma_{F}\right)\tau +\frac{1}{2}\xi^{2}\int_{t}^{T}\omega^{2}\left(u\right)du\right]}{\sqrt{\lambda^{2}\tau +\xi^{2}\int_{t}^{T}\omega^{2}\left(u\right)du}},\\ d_{2}^{\left(3\right)}=d_{1}^{\left(3\right)}-\sqrt{\lambda^{2}\tau +\xi^{2}\int_{t}^{T}\omega^{2}\left(u\right)du}\;. \end{array}$$

*Proof*. By martingale pricing theory and Girsanov theorem, we have

$$\begin{array}{c} V_{3}\left(S\left(t\right),F\left(t\right),\omega \left(t\right),\lambda ,\xi ,\rho ,\sigma_{F}\right)\\ =e^{-r_{d}\tau }E^{Q}\left[F_{0}\;\text{max}\;\left\{S\left(T\right)-K_{f},0\right\}|F_{t}\right]\\ =e^{-r_{d}\tau }F_{0}E^{Q}\left[S\left(T\right)I_{\left\{K_{f}<S\left(T\right)\right\}}|F_{t}\right]-e^{-r_{d}\tau }F_{0}K_{f}E^{Q}\left[I_{\left\{K_{f}<S\left(T\right)\right\}}|F_{t}\right]\\ =e^{-r_{d}\tau }F_{0}S\left(t\right)e^{(r_{f}-\rho \lambda \sigma_{F})\tau }E^{Q_{1}}\left[I_{\left\{K_{f}<S\left(T\right)\right\}}|F_{t}\right]-e^{-r_{d}\tau }F_{0}K_{f}E^{Q}\left[I_{\left\{K_{f}<S\left(T\right)\right\}}|F_{t}\right]\\ =F_{0}e^{-r_{d}\tau }\left[S\left(t\right)e^{(r_{f}-\rho \lambda \sigma_{F})\tau }\Phi \left(d_{1}^{\left(3\right)}\right)-K_{f}\Phi \left(d_{2}^{\left(3\right)}\right)\right], \end{array}$$ (16)

where

$$\begin{array}{c} d_{1}^{\left(3\right)}=\frac{\text{ln}\;\frac{S(t)}{K_{f}}+\left[\left(r_{f}+\frac{1}{2}\lambda^{2}-\rho \lambda \sigma_{F}\right)\tau +\frac{1}{2}\xi^{2}\int_{t}^{T}\omega^{2}\left(u\right)du\right]}{\sqrt{\lambda^{2}\tau +\xi^{2}\int_{t}^{T}\omega^{2}\left(u\right)du}},\\ d_{2}^{\left(3\right)}=d_{1}^{\left(3\right)}-\sqrt{\lambda^{2}\tau +\xi^{2}\int_{t}^{T}\omega^{2}\left(u\right)du}\;. \end{array}$$

**Theorem 4**. *Suppose the underlying foreign asset is an imperfectly liquid stock S*(*t*) *defined by* Eq (1), *then the time*-*t* *price of the equity-linked foreign exchange call option at maturity T with payoff* *S*(*T*) max{*F*(*T*) − *K*_*F*_, 0} *is*

$$V_{4}\left(S\left(t\right),F\left(t\right),\lambda ,\rho ,\sigma_{F}\right)=S_{t}\left[F\left(t\right)\Phi \left(d_{1}^{\left(4\right)}\right)-K_{F}e^{(r_{f}-r_{d}-\rho \lambda \sigma_{F})\tau }\Phi \left(d_{2}^{\left(4\right)}\right)\right],$$ (17)

*where F*_0_ *is the predetermined fixed exchange rate*, *τ* = *T* − *t*, *and* *ω*(*t*) *is the liquidity level*, λ, *ξ* *are defined as previously, and*

$$\begin{array}{c} d_{1}^{\left(4\right)}=\frac{\text{ln}\;\frac{F(t)}{K_{F}}+\left[\left(r_{d}-r_{f}+\frac{1}{2}\sigma_{F}^{2}+\rho \lambda \sigma_{F}\right)\tau \right]}{\sigma_{F}\sqrt{\tau }},\\ d_{2}^{\left(4\right)}=d_{1}^{\left(4\right)}-\sigma_{F}\sqrt{\tau }\;. \end{array}$$

*Proof*. By martingale pricing theory, we obtain

$$\begin{array}{cc} V_{4}\left(S\left(t\right),F\left(t\right),\lambda ,\rho ,\sigma_{F}\right) & =e^{-r_{d}\tau }E^{Q}\left[S\left(T\right)\;\text{max}\;\left\{F\left(T\right)-K_{F},0\right\}|F_{t}\right]\\ & =e^{-r_{d}\tau }E^{Q}\left[S\left(t\right)e^{(r_{f}-\rho \lambda \sigma_{F})\tau }\frac{dQ_{1}}{dQ}\;\text{max}\;\left\{F\left(T\right)-K_{F},0\right\}|F_{t}\right]\\ & =S\left(t\right)e^{(r_{f}-r_{d}-\rho \lambda \sigma_{F})\tau }E^{Q_{1}}\left[F\left(T\right)I_{\left\{K_{F}<F\left(T\right)\right\}}|F_{t}\right]\\ & \;\;\;-S\left(t\right)e^{(r_{f}-r_{d}-\rho \lambda \sigma_{F})\tau }E^{Q_{1}}\left[K_{F}I_{\left\{K_{F}<F\left(T\right)\right\}}|F_{t}\right] \end{array}$$ (18)

Under measure $Q_{1}$ defined by

$$\begin{array}{cc} \frac{dQ_{1}}{dQ}= & \text{exp}\{-\frac{1}{2}\int_{t}^{T}\left(\xi^{2}\omega^{2}\left(u\right)+\lambda^{2}\right)du+\int_{t}^{T}\sqrt{\xi^{2}\omega^{2}\left(u\right)+\lambda^{2}\left(1-\rho^{2}\right)}d{\tilde{W}}^{Q}\left(u\right)\\ & \;\;\;\;\;\;\;\;+\int_{t}^{T}\rho \lambda dW_{F}^{Q}\left(t\right)\}, \end{array}$$

we derive

$$d\;\text{ln}\;F(t)=\left(r_{d}-r_{f}-\frac{1}{2}\sigma_{F}^{2}+\rho \lambda \sigma_{F}\right)dt+\sigma_{F}dW_{F}^{Q_{1}}\left(t\right).$$

Denote the equivalent martingale measure by *Q*_2_ defined by

$$\begin{array}{c} \frac{dQ_{2}}{dQ_{1}}=\text{exp}\left\{-\frac{1}{2}\sigma_{F}^{2}t+\sigma_{F}W_{F}^{Q_{1}}\left(t\right)\right\}. \end{array}$$

According to the Girsanov theorem and probability theory, the first term on the right side of Eq (18) is rewritten by

$$\begin{array}{cc} S\left(t\right)e^{(r_{f}-r_{d}-\rho \lambda \sigma_{F})\tau }E^{Q_{1}}\left[F\left(T\right)I_{\left\{K_{F}<F\left(T\right)\right\}}|F_{t}\right] & =S\left(t\right)F\left(t\right)E^{Q_{1}}\left[\frac{dQ_{2}}{dQ_{1}}I_{\left\{K_{F}<F\left(T\right)\right\}}|F_{t}\right]\\ & =S\left(t\right)F\left(t\right)E^{Q_{2}}\left[I_{\left\{K_{F}<F\left(T\right)\right\}}|F_{t}\right]\\ & =S\left(t\right)F\left(t\right)\Phi \left(d_{1}^{\left(4\right)}\right), \end{array}$$

where

$$d_{1}^{\left(4\right)}=\frac{\text{ln}\;\frac{F(t)}{K_{F}}+\left[\left(r_{d}-r_{f}+\frac{1}{2}\sigma_{F}^{2}+\rho \lambda \sigma_{F}\right)\tau \right]}{\sigma_{F}\sqrt{\tau }}.$$

Similarly, the second term on the right side of Eq (18) is rewritten by

$$S\left(t\right)e^{(r_{f}-r_{d}-\rho \lambda \sigma_{F})\tau }E^{Q_{1}}\left[K_{F}I_{\left\{K_{F}<F\left(T\right)\right\}}|F_{t}\right]=S\left(t\right)e^{(r_{f}-r_{d}-\rho \lambda \sigma_{F})\tau }K_{F}\Phi \left(d_{2}^{\left(4\right)}\right),$$

where $d_{2}^{\left(4\right)}=d_{1}^{\left(4\right)}-\sigma_{F}\sqrt{\tau }$.

Therefore, the price of the equity-linked foreign exchange call option is

$$\begin{array}{c} V_{4}\left(S\left(t\right),F\left(t\right),\lambda ,\rho ,\sigma_{F}\right)=S_{t}\left[F\left(t\right)\Phi \left(d_{1}^{\left(4\right)}\right)-K_{F}e^{(r_{f}-r_{d}-\rho \lambda \sigma_{F})\tau }\Phi \left(d_{2}^{\left(4\right)}\right)\right]. \end{array}$$

Theorems (1)–(4) provide the theoretical pricing models to study the impact of stock liquidity on quanto options in an imperfectly liquid market. For better applying these theoretical models in practice, the precise estimation of unknown parameters is still required. As mentioned before, the main focus of this paper is to propose an approach to estimating model parameters and performing posterior inference on the quanto option price.

## 3 Posterior inference on the quanto option pricing model

Due to the lack of market data on quanto options, it is difficult to estimate the parameters of the quanto option pricing model. The accuracy of parameter estimation directly affects the performance of option pricing models. In this section, we propose a posterior inferential method to conduct statistical inferences on unknown parameters and the quanto option price.

### 3.1 Posterior inference on unknown parameters

Denote $x_{t}=\text{ln}\;\frac{S(t)}{S(t-1)}$ and $y_{t}=\text{ln}\;\frac{F(t)}{F(t-1)}$ as the continuously compounded returns. For simplicity, we let *ω*(*t*) = *ω*_*t*_. Under the risk-neutral measure Q, from Eqs (4) and (5), we derive the joint probability density

$$\begin{array}{c} p(x_{t},y_{t}|\lambda ,\xi ,\rho ,\sigma_{F},\omega_{t-1})\\ =\frac{1}{2\pi \sigma_{F}\sqrt{\xi^{2}\omega_{t-1}^{2}+\lambda^{2}}\sqrt{1-\rho^{2}}}\\ \;\;\times \text{exp}\;\{-\frac{1}{2(1-\rho^{2})}[\frac{{\left(x_{t}-r_{f}+\rho \lambda \sigma_{F}+\frac{1}{2}\xi^{2}\omega_{t-1}^{2}+\frac{1}{2}\lambda^{2}\right)}^{2}}{\xi^{2}\omega_{t-1}^{2}+\lambda^{2}}+\frac{{\left(y_{t}-r_{d}+r_{f}+\frac{1}{2}\sigma_{F}^{2}\right)}^{2}}{\sigma_{F}^{2}}\\ \;\;\;\;\;\;\;\;\;\;\;\;-\frac{2\rho \left(x_{t}-r_{f}+\rho \lambda \sigma_{F}+\frac{1}{2}\xi^{2}\omega_{t-1}^{2}+\frac{1}{2}\lambda^{2}\right)\left(y_{t}-r_{d}+r_{f}+\frac{1}{2}\sigma_{F}^{2}\right)}{\sigma_{F}\sqrt{\xi^{2}\omega_{t-1}^{2}+\lambda^{2}}}]\}. \end{array}$$

Denote the return observations by *X* = (*x*_1_, *x*_2_, ⋯, *x*_*T*_)′ and *Y* = (*y*_1_, *y*_2_, ⋯, *y*_*T*_)′, then the likelihood function is given by

$$\begin{array}{c} L(X,Y|\lambda ,\xi ,\rho ,\sigma_{F},\omega )\\ =\prod_{t=1}^{T}\{\frac{1}{2\pi \sigma_{F}\sqrt{\xi^{2}\omega_{t-1}^{2}+\lambda^{2}}\sqrt{1-\rho^{2}}}\\ \;\;\times \text{exp}\;\{-\frac{1}{2(1-\rho^{2})}[\frac{{\left(x_{t}-r_{f}+\rho \lambda \sigma_{F}+\frac{1}{2}\xi^{2}\omega_{t-1}^{2}+\frac{1}{2}\lambda^{2}\right)}^{2}}{\xi^{2}\omega_{t-1}^{2}+\lambda^{2}}+\frac{{\left(y_{t}-r_{d}+r_{f}+\frac{1}{2}\sigma_{F}^{2}\right)}^{2}}{\sigma_{F}^{2}}\\ \;\;\;\;\;\;\;\;\;\;\;\;-\frac{2\rho \left(x_{t}-r_{f}+\rho \lambda \sigma_{F}+\frac{1}{2}\xi^{2}\omega_{t-1}^{2}+\frac{1}{2}\lambda^{2}\right)\left(y_{t}-r_{d}+r_{f}+\frac{1}{2}\sigma_{F}^{2}\right)}{\sigma_{F}\sqrt{\xi^{2}\omega_{t-1}^{2}+\lambda^{2}}}]\}\}, \end{array}$$ (19)

where liquidity levels *ω* = (*ω*_0_, *ω*_1_, ⋯, *ω*_*T*−1_)′ are described by the liquidity measure RDV defined before.

The prior distribution represents the beliefs or assumptions about the parameters before we observe any data. The choice of prior distribution can have a significant impact on the results of Bayesian estimation. It can be chosen based on prior knowledge or empirical evidence. Common choices include uniform, normal, and exponential distributions. Based on the empirical evidence from existing literature, we consider truncated normal distribution as the prior distribution for parameters λ, *ξ*, and take uniform distribution *U* ∈ (−1, 1) as the prior distribution for correlation coefficient *ρ*. In addition, we take the noninformative prior for parameter *σ*_*F*_, i.e., $p\left(\sigma_{F}\right)\propto \frac{1}{\sigma_{F}}$. Supposing they are independent of each other. Thus, the joint prior probability density is represented by

$$p\left(\lambda ,\xi ,\sigma_{F},\rho \right)=f_{N}\left(\lambda \right)I_{\left\{\lambda >0\right\}}\cdot f_{N}\left(\xi \right)I_{\left\{\xi >0\right\}}\cdot p\left(\sigma_{F}\right)\cdot U_{-1<\rho <1},$$ (20)

where *f*_*N*_(⋅) denotes the probability density for standard normal distribution.

Based on Bayesian theorem, the kernel of the joint posterior probability density is given by

$$\begin{array}{c} p\left(\lambda ,\xi ,\sigma_{F},\rho |X,Y,\omega \right)\propto p\left(\lambda ,\xi ,\sigma_{F},\rho \right)L\left(X,Y|\lambda ,\xi ,\sigma_{F},\rho ,\omega \right)\\ \propto \frac{1}{\sigma_{\lambda }}e^{-\frac{{\left(\lambda -\mu_{\lambda }\right)}^{2}}{2\sigma_{\lambda }^{2}}}I_{\left\{\lambda >0\right\}}\times \frac{1}{\sigma_{\xi }}e^{-\frac{{\left(\xi -\mu_{\xi }\right)}^{2}}{2\sigma_{\xi }^{2}}}I_{\left\{\xi >0\right\}}\times \frac{1}{\sigma_{F}}\\ \;\;\times \prod_{t=1}^{T}\{\frac{1}{\sigma_{F}\sqrt{\xi^{2}\omega_{t-1}^{2}+\lambda^{2}}\sqrt{1-\rho^{2}}}\\ \times \;\text{exp}\;\{-\frac{1}{2(1-\rho^{2})}[\frac{{\left(x_{t}-r_{f}+\rho \lambda \sigma_{F}+\frac{1}{2}\xi^{2}\omega_{t-1}^{2}+\frac{1}{2}\lambda^{2}\right)}^{2}}{\xi^{2}\omega_{t-1}^{2}+\lambda^{2}}+\frac{{\left(y_{t}-r_{d}+r_{f}+\frac{1}{2}\sigma_{F}^{2}\right)}^{2}}{\sigma_{F}^{2}}\\ \;\;\;\;\;\;\;\;\;\;-\frac{2\rho \left(x_{t}-r_{f}+\rho \lambda \sigma_{F}+\frac{1}{2}\xi^{2}\omega_{t-1}^{2}+\frac{1}{2}\lambda^{2}\right)\left(y_{t}-r_{d}+r_{f}+\frac{1}{2}\sigma_{F}^{2}\right)}{\sigma_{F}\sqrt{\xi^{2}\omega_{t-1}^{2}+\lambda^{2}}}]\}\}, \end{array}$$ (21)

where *μ*_λ_, *σ*_λ_, *μ*_*ξ*_, and *σ*_*ξ*_ are hyperparameters of prior distributions for λ and *ξ*, respectively. We typically determine the values of hyperparameters through two steps. **Step 1**: We consider the noninformative prior for parameters λ, and *ξ*. By Bayesian formula, we obtain the posterior distributions for λ, and *ξ* based on the sample information. **Step 2**: Based on the posterior distributions obtained in Step 1, we perform statistical inference on λ, and *ξ*, including the mean, standard deviation, kernel density, etc. These estimation results are further treated as the prior information for choosing the values of the hyperparameters.

By conditional probability formula, the kernels of the fully conditional posterior probability densities are given by

$$\begin{array}{c} p(\lambda |\xi ,\sigma_{F},\rho ,X,Y,\omega )\\ \propto \frac{1}{\sigma_{\lambda }}e^{-\frac{{\left(\lambda -\mu_{\lambda }\right)}^{2}}{2\sigma_{\lambda }^{2}}}I_{\left\{\lambda >0\right\}}\times \prod_{t=1}^{T}\frac{1}{\sqrt{\xi^{2}\omega_{t-1}^{2}+\lambda^{2}}}\\ \;\times \text{exp}\;\{-\frac{1}{2(1-\rho^{2})}\sum_{t=1}^{T}[\frac{{\left(x_{t}-r_{f}+\rho \lambda \sigma_{F}+\frac{1}{2}\xi^{2}\omega_{t-1}^{2}+\frac{1}{2}\lambda^{2}\right)}^{2}}{\xi^{2}\omega_{t-1}^{2}+\lambda^{2}}\\ \;\;\;\;\;\;\;\;\;\;\;-\frac{2\rho \left(x_{t}-r_{f}+\rho \lambda \sigma_{F}+\frac{1}{2}\xi^{2}\omega_{t-1}^{2}+\frac{1}{2}\lambda^{2}\right)\left(y_{t}-r_{d}+r_{f}+\frac{1}{2}\sigma_{F}^{2}\right)}{\sigma_{F}\sqrt{\xi^{2}\omega_{t-1}^{2}+\lambda^{2}}}]\}, \end{array}$$ (22)

$$\begin{array}{c} p(\xi |\lambda ,\sigma_{F},\rho ,X,Y,\omega )\\ \propto \frac{1}{\sigma_{\xi }}e^{-\frac{{\left(\xi -\mu_{\xi }\right)}^{2}}{2\sigma_{\xi }^{2}}}I_{\left\{\xi >0\right\}}\times \prod_{t=1}^{T}\frac{1}{\sqrt{\xi^{2}\omega_{t-1}^{2}+\lambda^{2}}}\\ \;\times \text{exp}\;\{-\frac{1}{2(1-\rho^{2})}\sum_{t=1}^{T}[\frac{{\left(x_{t}-r_{f}+\rho \lambda \sigma_{F}+\frac{1}{2}\xi^{2}\omega_{t-1}^{2}+\frac{1}{2}\lambda^{2}\right)}^{2}}{\xi^{2}\omega_{t-1}^{2}+\lambda^{2}}\\ \;\;\;\;\;\;\;\;\;\;\;-\frac{2\rho \left(x_{t}-r_{f}+\rho \lambda \sigma_{F}+\frac{1}{2}\xi^{2}\omega_{t-1}^{2}+\frac{1}{2}\lambda^{2}\right)\left(y_{t}-r_{d}+r_{f}+\frac{1}{2}\sigma_{F}^{2}\right)}{\sigma_{F}\sqrt{\xi^{2}\omega_{t-1}^{2}+\lambda^{2}}}]\}, \end{array}$$ (23)

$$\begin{array}{c} p(\sigma_{F}|\lambda ,\xi ,\rho ,X,Y,\omega )\\ \propto {\sigma_{F}}^{-(T+1)}\times \text{exp}\;\{-\frac{1}{2(1-\rho^{2})}\sum_{t=1}^{T}[\frac{{\left(x_{t}-r_{f}+\rho \lambda \sigma_{F}+\frac{1}{2}\xi^{2}\omega_{t-1}^{2}+\frac{1}{2}\lambda^{2}\right)}^{2}}{\xi^{2}\omega_{t-1}^{2}+\lambda^{2}}\\ \;\;\;\;\;\;\;\;\;\;\;+\frac{{\left(y_{t}-r_{d}+r_{f}+\frac{1}{2}\sigma_{F}^{2}\right)}^{2}}{\sigma_{F}^{2}}\\ \;\;\;\;\;\;\;\;\;\;\;-\frac{2\rho \left(x_{t}-r_{f}+\rho \lambda \sigma_{F}+\frac{1}{2}\xi^{2}\omega_{t-1}^{2}+\frac{1}{2}\lambda^{2}\right)\left(y_{t}-r_{d}+r_{f}+\frac{1}{2}\sigma_{F}^{2}\right)}{\sigma_{F}\sqrt{\xi^{2}\omega_{t-1}^{2}+\lambda^{2}}}]\}, \end{array}$$ (24)

and

$$\begin{array}{c} p(\rho |\lambda ,\xi ,\sigma_{F},X,Y,\omega )\\ \propto {\left(1-\rho^{2}\right)}^{-\frac{T}{2}}\times \text{exp}\;\{-\frac{1}{2(1-\rho^{2})}\sum_{t=1}^{T}[\frac{{\left(x_{t}-r_{f}+\rho \lambda \sigma_{F}+\frac{1}{2}\xi^{2}\omega_{t-1}^{2}+\frac{1}{2}\lambda^{2}\right)}^{2}}{\xi^{2}\omega_{t-1}^{2}+\lambda^{2}}\\ \;\;\;\;\;\;\;\;\;\;\;+\frac{{\left(y_{t}-r_{d}+r_{f}+\frac{1}{2}\sigma_{F}^{2}\right)}^{2}}{\sigma_{F}^{2}}\\ \;\;\;\;\;\;\;\;\;\;\;-\frac{2\rho \left(x_{t}-r_{f}+\rho \lambda \sigma_{F}+\frac{1}{2}\xi^{2}\omega_{t-1}^{2}+\frac{1}{2}\lambda^{2}\right)\left(y_{t}-r_{d}+r_{f}+\frac{1}{2}\sigma_{F}^{2}\right)}{\sigma_{F}\sqrt{\xi^{2}\omega_{t-1}^{2}+\lambda^{2}}}]\}. \end{array}$$ (25)

We are noting that the fully conditional posterior probability densities are more complex. Thus, we propose a random walk chain Metropolis-Hastings algorithm to generate samples from the fully conditional posterior densities. Then, by Monte Carlo method, we perform statistical inference on parameters on the foundation of posterior samples λ^*j*^, *ξ*^*j*^, $\sigma_{F}^{j}$ and *ρ*^*j*^, *j* = 1, 2, ⋯, *N*. The inferential results consider the market data of stock prices and the exchange rate simultaneously, which is helpful for the parameter calibration of the quanto option pricing model.

### 3.2 Posterior inference on the quanto option price

In this section, we further perform statistical inference on the quanto option price based on the posterior estimations of the model parameters. From Theorems (1)–(4), we notice that the quanto option price is adjusted depending on the liquidity level *ω*(*t*), the sensitivity *ξ*, the correlation coefficient *ρ*, the volatility λ, and *σ*_*F*_.

Given the values of *S*(*t*), *F*(*t*), *ω*(*t*) and *τ* at time *t*, the quanto option price with liquidity risk *V*(*S*(*t*), *F*(*t*), *ω*(*t*), λ, *ξ*, *ρ*, *σ*_*F*_) is the function of parameters λ, *ξ*, *ρ*, and *σ*_*F*_ from a mathematical perspective. Based on posterior samples λ^*j*^, *ξ*^*j*^, *ρ*^*j*^ and $\sigma_{F}^{j}$, $V^{j}\left(S\left(t\right),F\left(t\right),\omega \left(t\right),\lambda^{j},\xi^{j},\rho^{j},\sigma_{F}^{j}\right)$ evaluated by Theorems (1)–(4) can be treated as the posterior samples of the quanto option price; refer to [23] for more details.

By Monte Carlo integration, the posterior expectation of quanto option price is

$$\begin{array}{c} E[V\left(S\left(t\right),F\left(t\right),\omega \left(t\right),\lambda ,\xi ,\rho ,\sigma_{F}\right)|X,Y]\\ ≃\frac{1}{N-n}\sum_{j=n+1}^{N}V^{j}\left(S\left(t\right),F\left(t\right),\omega \left(t\right),\lambda^{j},\xi^{j},\rho^{j},\sigma_{F}^{j}\right). \end{array}$$

Furthermore, we can conduct statistical inference on any posterior moment we need. Unlike traditional methods which usually provide only a point estimate, the proposed method provides more statistical characteristics about quanto option prices from a probabilistic perspective. These statistical characteristics can provide more information for investors with different risk preferences to make better decisions.

## 4 Numerical analysis

In this section, we conduct the numerical analysis of the quanto option pricing model with liquidity risk under the Bayesian framework. Li et al. [10] studied the effect of stock liquidity on the quanto option price. They demonstrated that the quanto option pricing model with liquidity adjustment can better fit the market price. However, the existing literature pays little attention to the parameter estimation for the quanto option model. This paper proposes a numerical algorithm to estimate the model parameters.

### 4.1 Metropolis-Hastings algorithm for posterior simulation

From the fully conditional posterior densities (22)–(25), we notice that the posterior densities are not standard forms. The MCMC algorithm is required to generate posterior samples for further statistical inferences on model parameters and the quanto option price. Gibbs sampling and Metropolis-Hastings (M-H) sampling are two commonly used MCMC algorithms. When posterior densities are standard, Gibbs sampling is often used to draw samples. Similarly to [28], we apply the random walk chain Metropolis-Hastings algorithm to generate samples from the posterior densities which are nonstandard. Let *θ* = (λ, *ξ*, *σ*_*F*_, *ρ*) be the vector of unknown parameters, and $\theta_{i}^{\left(j\right)}$ denotes the sample of the *i*th element in vector *θ* at iteration *j* generated by following algorithm:

1. Draw a proposal $\theta_{i}^{*}$ from normal distribution $N(\theta_{i}^{\left(j-1\right)},\sigma_{\theta_{i}}^{2})$ with mean $\theta_{i}^{\left(j-1\right)}$ and variance $\sigma_{\theta_{i}}^{2}$.
2. Draw an observation *u* from uniform distribution *U*[0, 1].
3. Compute the acceptance probability $\tilde{\alpha }\left(\theta_{i}^{\left(j-1\right)},\theta_{i}^{*}\right)=\text{min}\;\{\frac{p(\theta_{i}^{*}|\theta_{-i}^{\left(j-1\right)},X,Y,\omega )}{p(\theta_{i}^{\left(j-1\right)}|\theta_{-i}^{\left(j-1\right)},X,Y,\omega },1\}$.
4. If $\tilde{\alpha }\left(\theta_{i}^{\left(j-1\right)},\theta_{i}^{*}\right)<u$, then $\theta_{i}^{\left(j\right)}=\theta_{i}^{*}$, otherwise $\theta_{i}^{\left(j\right)}=\theta_{i}^{\left(j-1\right)}$.

Where $\theta_{-i}^{\left(j-1\right)}$ is the sample generated in the last step for elements in vector *θ* excluding the *i*th one.

Meanwhile, to evaluate the efficiency of the proposed method, we employed the nonlinear optimization (NOP) method introduced in [10, 15] to estimate the model parameters for quanto option pricing. The objective function of the NOP method is to minimize the sum of the squared price differences between the model and market prices of all available options. The parameter estimations can be obtained by the following procedure:

$$\begin{array}{c} SSE\left(t\right)=\underset{\lambda ,\xi ,\rho ,\sigma_{F}}{\text{min}}\;\sum_{k=1}^{Z}{\left[V_{market}^{k}\left(t,\tau ,K\right)-V_{model}^{k}\left(t,\tau ,K,\lambda ,\xi ,\rho ,\sigma_{F}\right)\right]}^{2}, \end{array}$$

where $V_{market}^{k}\left(t,\tau ,K\right)$ represents the market price of the quanto option, and $V_{model}^{k}\left(t,\tau ,K,\lambda ,\xi ,\rho ,\sigma_{F}\right)$ is the model price evaluated by theoretical models. λ, *ξ*, *ρ*, and *σ*_*F*_ are the unknown parameters to be estimated.

### 4.2 Application to market data

We consider such a quanto option contract in which an investor in Canada invests in the European call option written on the Facebook In. (FB) stock traded in America. The investor is exposed to the market risk of FB stock price and the exchange rate between US dollars and Canadian dollars (USD/CAD).

**Fig 1:**
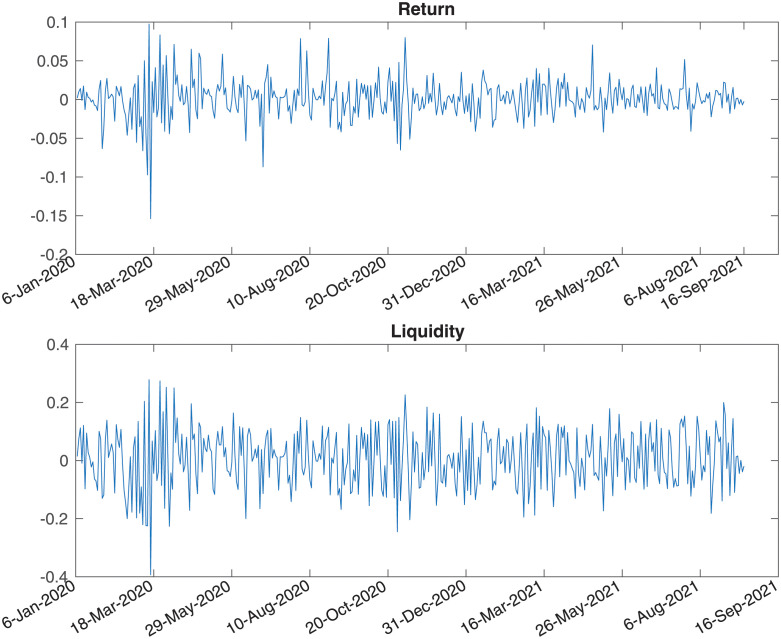
The returns and the liquidity levels of FB stock.

After COVID-19 in 2020, the panic selling of the market caused a sharp drop in the stock market. In addition, the liquidity change caused by the tightening of monetary policy is also an important reason for the withdrawal of the stock market. Therefore, we obtained the market data of FB stock prices and USD/CAD exchange rate from the Yahoo finance website from 6 January 2020 to 17 September 2021. During the period, we found that the stock market suffered from the disturbance of COVID-19 and the global liquidity crunch. The risk-free interest rate is obtained by the LIBOR rate, and the FB stock has zero dividend during the sample period. Stock liquidity level is measured by RDV proxy defined before. Fig 1 shows the returns and the liquidity levels of FB stock under the liquidity measure RDV. From Fig 1, we notice that stock returns and liquidity have changed significantly during the sample period indicating that there is indeed a correlation between asset prices and market liquidity.

Firstly, we perform statistical inference on unknown parameters based on the posterior distributions (22)–(25) by the MCMC numerical algorithm. The random walk chain Metropolis-Hastings algorithm described in Section 4.1 is conducted 50000 times and discards the initial 25000 samples to remove the impact of initial values on estimations. The convergence of the Markov chain is received according to Geweke’s convergence diagnostic [29].

Furthermore, we investigate the out-of-sample pricing performance of the proposed model by comparing the model price with the market price. Although the market price data of the quanto option is unavailable, we adopt a similar method in [10] to construct the quanto option price as the benchmark by the market price of the European call option written on FB stock and the USD/CAD exchange rate. Denote by *Qcall*(*t*, *K*, *T*) the market price of the quanto option, then we have

Qcall(t,K,T)=F(t)Call(t,K,T),

where *F*(*t*) is the USD/CAD exchange rate and *Call*(*t*, *K*, *T*) is the market price of the European call option written on FB stock.

**Fig 2:**
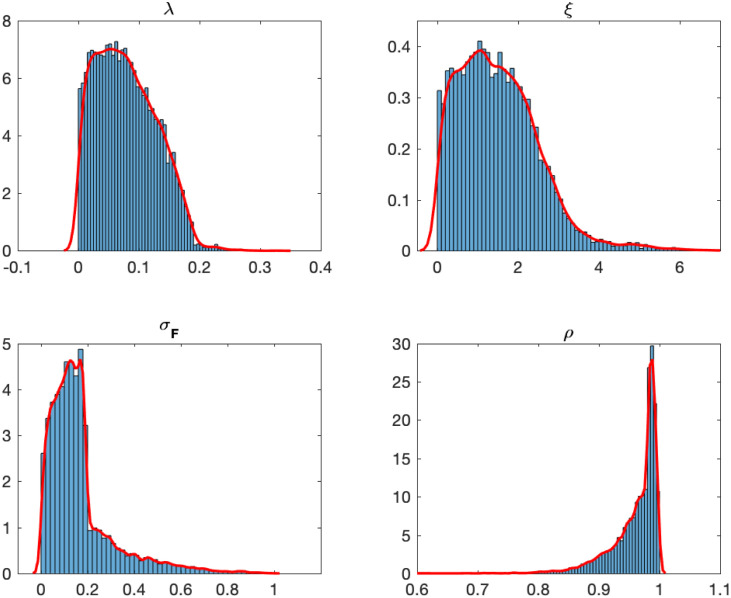
Posterior histogram and posterior kernel density for parameters under liquidity measure RDV.

Table 1 shows the posterior estimations of model parameters, including the posterior mean (Mean), posterior standard deviation (Std.Dev.), and MCMC convergence diagnostics. Denote the numerical standard errors by ‘NSE’ which shows the estimation accuracy. The column marked ‘CD’ introduced by [29] is used to judge the convergence of the Markov chain. A common rule is to conclude that convergence of the MCMC algorithm has been achieved if ‘CD’ is less than 1.96 in absolute value for all parameters. Table 1 indicates that convergence of the MCMC algorithm has been achieved. The last column is the 99% highest posterior probability density interval (HPDI) for parameters. Fig 2 shows the posterior probability histogram and kernel density for parameters under the liquidity measure RDV.

**Table 1:** Posterior results using Bayesian method and NOP estimates for quanto option model.

Method	Parameter	Mean	Std.Dev.	NSE	CD	99%HPDI
Bayesian	λ	0.0792	0.0493	0.0005	-1.2136	[0.0017, 0.1892]
	*ξ*	1.5142	1.0169	0.0101	-0.8077	[0.0379, 4.8681]
	*σ*_*F*_	0.1645	0.1425	0.0015	0.3871	[0.0059, 0.7079]
	*ρ*	0.9607	0.0386	0.0004	-0.7408	[0.8230, 0.9987]
NOP	λ	0.3034	-	-	-	-
	*ξ*	3.2755	-	-	-	-
	*σ*_*F*_	-	-	-	-	-
	*ρ*	-	-	-	-	-

We can see that the Bayesian method can provide more estimation results for model parameters. However, the traditional NOP method, which relies on a large number of market data of the quanto option price, only provides a point estimation. We can not obtain the estimation results of parameters *σ*_*F*_ and *ρ* by using the NOP method. Moreover, under the Bayesian framework, parameter uncertainty and the randomness of the correlation coefficient *ρ* are considered in the option pricing model. From posterior probability densities (22)–(25), we note that the inferential results consider the market data of stock prices and the exchange rate simultaneously, which is helpful for the parameter calibration of the quanto option pricing model.

Similarly to [10], we take the floating exchange rate foreign equity quanto call option stuck in foreign currency as an example. We illustrate how to conduct posterior inference on the quanto option pricing model using the Bayesian method combined with Monte Carlo numerical algorithm.

Now we evaluate the quanto option with time to maturity *τ* = 28, 42, 63, 120 days, respectively. Due to the limitation of the paper space, we only present the pricing results for the quanto option with a maturity of 28 days. Based on the posterior samples of parameters λ, *ξ*, *σ*_*F*_ and *ρ*, we conduct posterior inference on the quanto option price combining *V*_1_(*S*(*t*), *F*(*t*), *ω*(*t*), λ, *ξ*) defined by Eq (6). Under the Bayesian framework, we can get the posterior mean, standard deviation, quantiles, the 99%HPDI, and the posterior kernel density for the option price. These posterior results provide more statistical characteristics about the option price from a probabilistic perspective for investors with different risk preferences to make better decisions. The proposed method considers the effect of parameter uncertainty and correlation coefficient randomness on the option price.

**Fig 3:**
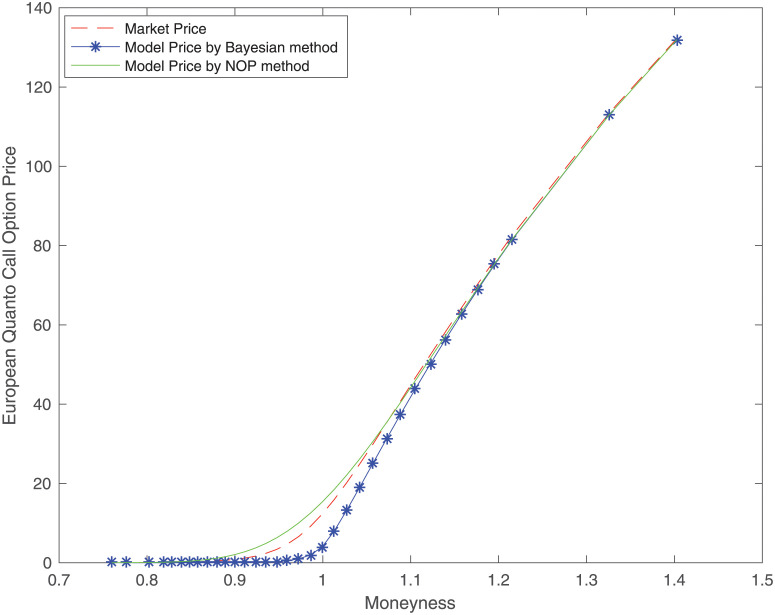
Quanto option price with liquidity adjustment by Bayesian method and NOP method.

To assess the pricing performance, we adopt the absolute percentage pricing errors as the evaluation criteria. The absolute percentage pricing errors are defined by the absolute difference between the market price and the model price over the market price. Under liquidity measure RDV, Table 2 shows the posterior estimations of the quanto option price evaluated by the Bayesian method and the corresponding model price evaluated by Eq (6) where the parameters are estimated by the NOP method. It reports the mean and standard deviation of the pricing errors for different moneyness categories, where moneyness is the stock price divided by the strike price. OTM, NTM, and ITM denote out-of-the-money, near-the-money, and in-the-money options, respectively. Table 2 shows that the standard deviation of pricing errors using the Bayesian method is 0.0304 and the standard deviation is 0.4414 using the NOP method. This indicates that the pricing performance of the Bayesian method is more stable than that of the NOP method for OTM options. Additionally, the means are 0.9790, 0.6312, and 0.0623 for different moneyness categories, indicating the pricing error using the proposed method is lower for in-the-money options. Fig 3 shows the market price of the quanto option and the corresponding model price evaluated by the Bayesian method and the NOP method, respectively.

**Table 2:** Out-of-sample pricing performance across different moneyness groups.

Moneyness	Bayesian method		NOP method	
	Mean	Std.Dev.	Mean	Std.Dev.
OTM	0.9790	0.0304	0.9427	0.4414
NTM	0.6312	0.2099	0.2773	0.1686
ITM	0.0623	0.0699	0.0151	0.0125

We notice that the model price can fit the market price well, indicating that the proposed method is feasible in pricing the quanto option with liquidity risk. The pricing performance for OTM options and ITM options is better than that for NTM options. For NTM options, the model price evaluated by the NOP method is bigger than the market price. However, the model price evaluated by the Bayesian method is smaller than the market price. This paper provides an approach to estimating the model parameters and performing posterior inference on the quanto option with liquidity risk.

## 5 Conclusion

Existing literature pays little attention to the parameter estimation for the quanto option pricing model with liquidity risk. Due to the lack of market data on quanto options, traditional estimation methods may not be applicable to the quanto option pricing model. Therefore, this paper proposes an approach to estimating the model parameters and performing posterior inference on the quanto option price under the Bayesian framework. First, we derive the theoretical pricing formula of the quanto option with liquidity risk. Second, based on the dynamics of the underlying stock price and the exchange rate process, we construct a likelihood function for performing posterior inference on model parameters. We provide a different perspective to estimate the correlation coefficient. Furthermore, we illustrate how to evaluate the quanto option with liquidity risk based on posterior densities by a random walk chain Metropolis-Hastings sampling algorithm. Finally, an empirical analysis is conducted to examine the pricing performance. The empirical results demonstrate the proposed method is feasible in pricing the quanto option with liquidity risk.

This paper provides an alternative approach to estimating the model parameters and performs posterior inference on the quanto option with liquidity risk. The proposed method also applies to other multi-asset option pricing models. For instance, the extensions on how to incorporate stock market liquidity and option market liquidity into option pricing models remain open in future studies.

## Supporting information

S1 Dataset (ZIP)
